# Efficacy of Losartan in the management of Post-Dialysis Euvolemic Hypertension (HELD-Trial): A Single-Blind Randomized Control Trial

**DOI:** 10.1038/srep36592

**Published:** 2016-12-06

**Authors:** Raja Ahsan Aftab, Amer Hayat Khan, Azreen Syazril Adnan, Syed Azhar Syed Sulaiman, Tahir Mehmood Khan

**Affiliations:** 1 Discipline of Clinical Pharmacy, School of Pharmaceutical Sciences Universiti Sains Malaysia, 11800 Penang, Malaysia; 2 CKD Resource Centre, School of Medical Sciences, Universiti Sains Malaysia, Kubang Kerian, 16150, Kelantan, Malaysia; 3 School of Pharmacy, Monash University, Bandar Sunway 45700, Selangor, Malaysia; 4 Department of Pharmacy, Abasyn University, Peshawar, Khyber Pakhtunkhwa, Pakistan

## Abstract

To assess the effectiveness of losartan 50 mg on post dialysis euvolemic hypertensive patients against standard antihypertensive pharmacotherapy. A multicentre, prospective, randomized, single-blind trial was conducted to assess the effect of losartan 50 mg every other day (EOD), once a morning (OM) among post-dialysis euvolemic hypertensive patients. Covariate-adaptive randomization was used to allocate participants to a standard or treatment arm, and they were followed up for eight weeks. Pre-, intra- and post-dialysis session blood pressure (BP) measurements were recorded along with any adverse events. A total of 88 patients were randomized into standard (n = 44) and treatment arms (n = 44) and were followed for a period of 8 weeks. In the standard group, the mean post-dialysis blood pressure dropped by 0.3 mmHg by the end of the 8^th^ week. However the treatment arm reported a drop of 2.4 mmHg of BP drop during the 8-week trial period. Analysis suggests that there was a significant difference in blood pressure readings at the end of 8 weeks among patients treated with losartan (P < 0.001). However, no such statistical association was observed in the standard arm (P 0.75). A slow, steady significant decline in post-dialysis BP was observed among euvolemic hypertensive patients that were treated with losartan 50 mg.

With annual mortality rates of 23%, hypertension is one of the most prevalent conditions reported among haemodialysis patients^1^,^2^. Since there is a significant variation in blood pressure of pre-, intra- and post-dialysis, the management of hypertension among haemodialysis patients presents different challenges. One of these is the method of blood pressure measurement. The extreme changes in blood volume often make it difficult to obtain a clear picture of the actual blood pressure in haemodialysis patients. Moreover, to further complicate the scenario, attaining an ideal targeted blood pressure range has often been an area of debate since the targeted blood pressure from guidelines is often difficult to achieve in clinical practice.

Hypertension in haemodialysis is multi-factorial and is not completely elucidated with volume overload. Renin angiotensin aldosterone system (RAAS) activation has been identified as one of the major contributors. Although patients may have a normal range of plasma rennin activity, its relation to exchangeable sodium has long been identified as associated with hypertension. Interestingly, the renin angiotensin system is activated in haemodialysis patients by the fact that renin is increased with haemodialysis ultra-filtration leading to hypertension^3^. Normally, volume overload and elevated blood pressure suppress the renin system, since this feedback mechanism is incomplete in chronic kidney disease (CKD) patients due to parenchymal renal injury and renovascular disease; CKD patients often have high blood pressure with normal or elevated renin levels^4^. Literature suggests that renin levels may be twice as high in hypertensive haemodialysis patients than normal haemodialysis patients, with a study reporting plasma renin activity increasing from 2.3 ± 0.5 ng/ml/hr at just before initiation of HD to 6.5 ± 1.3 ng/ml/hr over an 8- to 10-year period among hypertensive haemodialysis patients^4^,^5^. Similar clinical data suggests kidney disease progression is related to aldosterone, as literature suggest elevated aldosterone levels were observed in patients with average creatinine clearance of <14 ml/min^6^. Even after complete decline of kidney function, these finding suggests that renin secretion continues.

Fluid balance is an integral part of the management of haemodialysis patients, to prevent under- or over-hydration, which are associated with cardiovascular and other complications^7^. A majority of fluid removal and maintenance of an ideal hydration status is targeted with patient dry weight, where dry weight is clinically determined by the lowest weight a patient can tolerate without intradialytic symptoms and hypotension in the absence of fluid overload^8^. In the assessment of volume status, a bioimpedance device or body composition monitor (BCM) provide the quantification analysis of excess extracellular volume through a comparison with a healthy population, thereby providing a reliable account of a patient’s dry weight and hydration status^9^. Significantly, volume-dependent components of hypertension can be corrected by appropriate volume removal. However, a proportion of haemodialysis patients experience elevated blood pressure despite achieving euvolemia and ideal dry weight^10^. Since there is a constant volume variation during haemodialysis session, there is a strong possibility of the activation of the RAAS system, leading to a rise in blood pressure even if the patient is euvolemic. Considering the importance of the RAAS system in euvolemic hypertensive patients, the role of drugs blocking the RAAS system needs further investigation. ARBs and Angiotensin-Converting Enzyme Inhibitors (ACEIs) are similar and both are often used during clinical practice for en stage renal failure disease (ESRD) patients. An observational study suggests an angiotensin receptor blocker (ARB), in combination with another antihypertensive medication (but not an ACEI), may have a beneficial effect on cardiovascular mortality among ESRD patients on dialysis^11^. Hence the current study is based on role of ARBs in managing hypertension among euvolemic haemodialysis patients.

## Method

The current study is a multicentre, prospective, randomised, parallel design, single-blind trial. The Hospital Universiti Sains Malaysia (HUSM), Kelantan, is the main research site. Patients from three private dialysis centres were also included in the current trial. The study protocols were approved by the ethical and research committee of Universiti Sains Malaysia (USM/JEPeM/15050173), and the trial was registered under Australian New Zealand Clinical Trials Registry (ACTRN12615001322527). The trial was registered on 2/12/2015, the first patient was enrolled on 10/12/2015, and the trial formally began on 8/02/2016. The study purpose was explained and informed consent was obtained before enrolling study participants. The study protocols met consolidated standard of reporting trials (CONSORT) guidelines. All study procedures were in accordance with national kidney foundation kidney disease outcome quality initiative (NKF KDOQI) practice guidelines for haemodialysis^12^. Based on cost-effectiveness and availability, and the opinion of a panel of experts, a decision was taken to use an ARB (Losartan) for the treatment group.

### Study participants

Post-dialysis euvolemic patients with systolic blood pressure >140 mmHg post dialysis, patients aged 30–80, patients undergoing dialysis for of at least 12 months, and patients willing to participate were included in the trial. Patients with amputations, pre-existing heart disease, neoplasm and cystic kidneys, patients treated with ARBs and patients with symptomatic, predialytic hypotension (SBP less than 110 mm Hg) or high blood pressure >200/100 mmHg were excluded from the study. Consent was taken from all the study participants. An appropriate time was given to patients for their consent to join the study. No patient was recruited for the study unless he/she had agreed to join the study without any external or internal pressure. Only the study participants were blinded during the course of study. All study participants underwent dialysis thrice a week. Hence all patients were regularly monitored for their blood pressure, adherence to medication and other study related parameters.

### Pre-screening

A body composition monitor device (BCM) by Fresenius (BCM 4BJA3641) was used for assessing the volume status of patients at the end of a dialysis session and was also used for estimating patients’ dry weight^13^,^14^. After assessing dry weight by BCM, all efforts were made to ensure patients achieved their dry weight at the end of dialysis sessions. Patients with systolic blood pressure >140/90 mmHg thirty minutes after the dialysis sessions underwent a volume assessment by a BCM device. The blood pressure (BP) readings were taken at sitting position by a haemodialysis staff nurse using a manual caliberated sphygmomanometer. Post-dialysis hypertensive patients (>140/90 mmHg) found euvolemic in three consecutive dialysis sessions were included in the trial. In addition, a regular BCM assessment for dry weight was done to ensure patients attained their specific dry weights and euvolemic states. Literature suggests that systolic blood pressure is constantly associated with cardiovascular adverse events^15^, which is why systolic blood pressure is considered as a marker for outcomes in the current trial.

### Randomization of study participants

In randomized controlled clinical trials, a balanced allocation of covariates is an essential component in ensuring valid treatment comparisons. Covariate-adaptive randomization has an advantage over other randomization techniques, in that it is able to achieve a balance over a large number of covariates when the sample size is small to medium^16^. Covariate- adaptive randomization was used for assigning all pre-screened participants who have agreed to participate in the study to the treatment or standard arm. On the basis pre-existing literature, covariates considered for the current study are age, gender, diabetes and year of dialysis that could affect our treatment outcomes hence to avoid this, all participants were randomized using covariate adaptive randomization that would devoid any influence of covariates on randomization^17^. Randomization of study participants was done by a computer programme to minimize the risk of selection bias and avoid any influence of the researcher, the prescriber or the participant in the selection of group or medication^18^. Furthermore, all enrolled patients underwent a minimum two-week wash-out period to avoid any bias in the study. During the wash-out period, patients, in consultation with a cardiologist and a nephrologists, were prescribed other hypertensive medication (except RAAS inhibitors) to maintain their blood pressure and to avoid any study bias. Upon the completion of the wash-out period the patients entered the regular trial phase.

### Standard and treatment arms

Both standard and treatment arms were randomized with equal number of patients. The standard arm patients received antihypertensive therapy (except RAAS inhibitors) including calcium channel blocker, diuretic, alpha and beta blockers, whereas the treatment arm patients were prescribed ARB (losartan) alone or in combination with standard antihypertensive pharmacotherapy. In both study arms, patients were allowed multiple antihypertensive pharmacotherapies to control blood pressure, upon discussion with a panel of experts, but only where entirely necessary to avoid any deterioration in a patient’s health. Finally, study intervention (losartan) and antihypertensive medication were given to the patients on a weekly basis by the principle investigator. A member of family was consented to monitor patient adherence at home whereas a pill check was performed once a week.

### Study End Point

**Figure 1:**
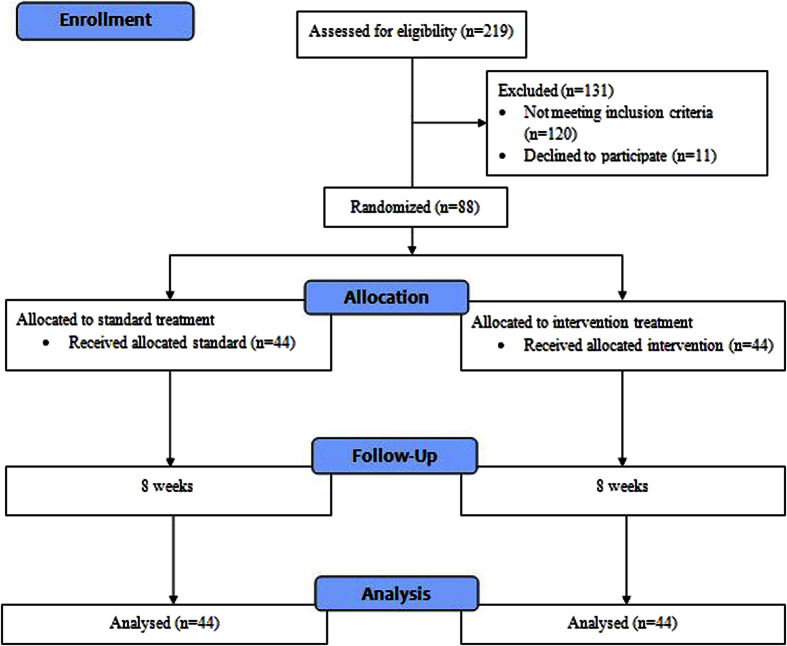
Study flow chart.

A medication dose of losartan 50 mg/day for three weeks as a test dose was initiated to note any incidence of hypotension. Upon satisfaction, patients were continued with 50 mg/day every day apart from dialysis days. A persistent >180 mmHg post-dialysis blood pressure despite adding three hypertensive agents resulted in dose titration. The dose titration was initiated with losartan 50 mg to 100 mg followed by non-RAAS antihypertensive agents for treatment group. For the standard group, the choice of dose titration was left to expert opinion^19^,^20^. The primary end point was achieving targeted post-dialysis blood pressure of <140/90 mmHg and maintaining this for four weeks, whereas the secondary end point was all causes of mortality. Figure 1 describes the study flow.

### Sample size and statistical analysis

The sample size for the current study was based on a statistical superiority trial (continuous data) design of a randomized control trial^21^.


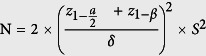


where, N = size per group; p = the response rate of standard treatment group; p 0 = the response rate of new drug treatment group; z = the standard normal deviate for a one or two sided x; d = the real difference between two treatment effect; a clinically acceptable margin; S = Polled standard deviation of both comparison groups. The sample size calculated from statistical superiority for randomized control trials is 35 for each arm, so altogether 70 euvolumic hypertensive euvolemic patients. Moreover a 25% dropout was also included, making total of 88 patients (44 in each arm).

Results were expressed as mean or percentage. Comparisons between treatment groups were made by using Wilcoxon tests after adjustment for the dynamic stratification variables (age, sex, years on dialysis, and diabetes). Cohen’s d test was applied to note the effect size. In addition, linear regression was applied to note any influence of patient characteristics on treatment outcome. This data is presented as hazard ratios and 95% confidence intervals. Statistical significance was set at *P* less than 0.05. All statistical calculations were performed using SPSS version 20.

## Results

All 88 patients [standard (n = 44) and treatment arm (n = 44)] completed the entire study and were followed for a period of 8 weeks. About 97% of the patients were ethnically Malay. Altogether, 45 male and 43 female patients were enrolled: 21 male and 24 female patients were randomized to the standard arm compared to 23 male and 20 female patients in the treatment arm. The mean age (standard 54.0 years, treatment 53.7 years) & median age (standard arm 54.5 years, treatment 55 years) age of both the arms were similar. 31 (70.5%) diabetic patients were randomized to the standard arm whereas 28 (63.6%) diabetic patients were randomized to the treatment arm (Table 1).

Since the treatment arm involved giving losartan to all 44 subjects, no ARB or ACE inhibitor was prescribed to the standard arm. A calcium channel blocker (n = 37, 84.1%) was the most common antihypertensive medication prescribed to the standard group followed by a diuretic (n = 16, 36.3%). Other medication included statins that was prescribed to 33 (75%) patients in the standard group and 32 (71.1%) patients in treatment group (Table 1).

### Primary and secondary outcome

The baseline blood pressure reading between both the groups were almost comparable. The mean baseline post-dialysis blood pressure of the standard arm was 157.5 (±14.3) mmHg compared to the treatment arm 156.3 (±13.4) mmHg. In the standard group, the mean post- dialysis blood pressure dropped by 0.3 mmHg by the end of 8 weeks, whereas in the treatment arm, the mean post-dialysis blood pressure reduced by 2.4 mmHg by the end of 8 weeks. Four (11.4%) patients in the first four weeks and 8 (22.8%) patients in the next four week in the treatment arm were able to achieve post-dialysis blood pressure of <140 mmHg but were not able to maintain it more than 3HD sessions. All study participants were maintained on losartan 50 mg only though out the coarse of study. There were no cases of mortality (Table 2).

**Figure 2:**
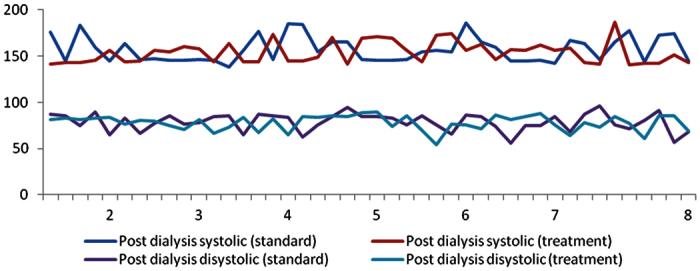
A comparison of post dialysis blood pressure readings after 8 weeks of patient follow up between standard and treatment arm. Post dialysis blood pressure comparison between standard and treatment group.

Figure 2 shows the comparison of post dialysis blood pressure readings after 8 weeks of patient follow up between standard and treatment arm. After eight weeks of study, the mean post-dialysis blood pressure (systolic) in the standard arm was 157.2 (±13.9) mmHg compared to 153.9 (±11.7) in the treatment arm.

Simple linear regression was performed to indicate patient-related factors that affected the study treatment outcome. Male (p 0.01) and diabetes (Standard p 0.01, Treatment p 0.01), patients on dialysis >5 years (p 0.01) and ex smokers (p 0.04) had a significant association with blood pressure control among study participants (Table 3).

A mean drop in systolic blood pressure is observed in both the study groups. However, the treatment group indicates a greater decline in mean systolic blood pressure compared to the standard group. Further analysis suggests that blood pressure readings of the treatment group were significantly different at the end of the 8-week period (p < 0.001), whereas the standard arm had a non-significant blood pressure (p 0.75) difference at the end of 8 weeks from the baseline blood pressure reading (Table 4).

### Adherence, drop out and adverse events

During the course of study, there were no cases of drop-out due to non-adherence to medication, adverse events or any other clinical scenario. Headaches were the most common ADR reported in the standard and treatment arms during HD session. Twelve (27.7%) patients reported ≤2 instants of headaches during the HD session in the standard group whereas 10 (22.7%) patients in the treatment group reported ≤2 and 2 (4.5%) reported 3–5 instants of headaches during HD sessions. There were no reported cases of hyperkalemia in either group (Table 5).

## Discussion

In the past, the emphasis has been on an overall BP reduction in haemodialysis patients. However, a “one-size-fits-all” approach for BP management may not be appropriate for all the patients on haemodialysis^22^. Similarly, the management of euvolemic hypertension among haemodialysis patients has often remained neglected. Blood pressure and volume control are critical to prevent mortality and morbidity among haemodialysis patients^23^,^24^.

The current study reports that the losartan (ARB) 50 mg together with other antihypertensive drugs lowered BP by 2.4 mmHg (0.17[−2.81, 9.89]) amongst euvolemic patients that were hypertensive (>140 mmHg) at the end of HD sessions. Our analysis also indicates that there was a statistically significant difference in systolic blood pressure from baseline and at the end of the 8-week study period among the treatment group. In a study by Iseki *et al*., <20% of study participants reached the study goal of <140/90 mmHg during 3.5 years of patient follow-up^25^. In the current study, 8 patients were able to achieve the targeted blood pressure of <140 mmHg but could not maintain it for more than three sessions. Ultimately, none of the patients were able to achieve the study end points. The current trial emphasis was first on volume management and then blood pressure control. Patients that are hypertensive despite achieving euvolemic state use renin inhibitors to neutralize any renin activity in the euvolemic state.

In a trial demonstrating the effects of telmisartan on haemodialysis patients, Huber *et al*. reported an initial significant decline in blood pressure followed by non-significant difference in blood pressure reading among both groups. The authors attribute these findings to a relatively small sample size and duration, and most importantly vast variability in blood pressure^26^. As in our study, blood pressure variability in some study subjects was one of the factors that could account for the initial decrease and rise in blood pressure in the standard arm. In both arms, no medications of any study participants were changed during the course of the study. However, patients in the treatment arms showed slow but progressive decline in post-dialysis systolic blood pressure.

National kidney foundation guidelines recommend a pre-dialysis blood pressure of <140/90 mmHg and a post-dialysis blood pressure of <130/80 mmHg as targeted BPs for haemodialysis patients. However, there are some concerns regarding the targeted blood pressures, since most of the data is largely manipulated from observational studies from non-ESRD patients. Hence, targeted blood pressures among haemodialysis patients remain unclear^27^. Davenport *et al*. report higher incidence of intradialytic hypotension in patients achieving post-dialysis BP targets^28^. In turn, intradialytic hypotension is associated with mortality^29^,^30^ thereby raising questions regarding the recommended post-dialysis blood pressure target range. Similarly studies indicate a U-shaped or reverse J-shaped relationship between systolic blood pressure and mortality among ESRD patients^31^,^32^. Hence, a targeted post-dialysis of <140 mmHg systolic blood pressure was adopted for the current study.

The current study also reports a drop in pre-dialysis blood pressure by 3.2/1 mmHg in the standard arm and 9/2.5 mmHg in the treatment arm during the 8-week study period. Studies have reported that lower (<120 mmHg) and higher pre-dialysis (>160 mmHg) systolic blood pressure is associated with high mortality rates^33^. The current trial focuses on renin inhibition among euvolemic patients, which is more activated at the end of HD sessions. Pre-dialysis blood pressure is largely associated with volume overload, so even though it is important it was discussed in the current study.

ARBs are usually well tolerated, though hyperkalemia is frequently encountered among Haemodialysis patients on RAAS inhibitors irrespective of medication used. Hence blood profiles of all study participants were performed to keep a track of potassium levels and other blood parameters. No cases of hyperkalemia were reported in the current study. Twelve of the patients in the standard arm and 10 patients in the treatment arm suffered headaches ≤2 times during their HD sessions. None of the patients suffered from post-dialysis hypotension. However, 13 treatment-arm patients and 11 standard-arm patients complained of leg cramps ≤2 times after dialysis sessions. Considering their post-dialysis blood pressure readings, leg cramps were more associated with dialysis procedure rather than the effect of antihypertensive medication.

### Limitation

A small sample size is one of the main limitations of this study; future studies should consider addressing a similar study question with a bigger population. In addition, there will be some concerns about generalizing the results of this study. The majority of the patients participated in this trial were Malays. Therefore the results of this study cannot be generalized for the whole population of Malaysia. Keeping in mind the principle of genetic influence, it might be possible that other ethnic groups, i.e. Chinese or Indians, might have different outcomes in comparison to Malays. Accurate BP measurement is fundamental to clinical practice and research, yet there is no consensus on which BP measurements to use and define hypertension in haemodialysis patients. Studies indicate that routine pre-dialysis or post-dialysis BP readings may overestimate BP^34^. A similar difference in measuring BP also exists, which is to take the BP reading in either the supine or sitting position, though the literature reported difference of these. Hence there is no consensus over the optimal method of BP measurement among haemodialysis patients^35^.

## Conclusion

The management of euvolemic hypertension among ESRD patients has rarely been investigated. The current study demonstrates the beneficial effects of losartan (ARB) in lowering the blood pressure of euvolemic hypertensive patients. The importance of accurate assessment and achieving clinical dry weight cannot be neglected in achieving a euvolemic state at the end of the dialysis session. Treating euvolemic hypertension as a separate entity would not only have benefits in lowering post-dialysis blood pressure, but has also proven to improve pre-dialysis blood pressure subjected to non-pharmacological treatment. Reduction in overall blood pressure would not only lead to an increase in overall survival rates but also improve the quality of life among ESRD patients.

## Additional Information

**How to cite this article**: Aftab, R. A. *et al*. Efficacy of Losartan in the management of Post-Dialysis Euvolemic Hypertension (HELD-Trial): A Single-Blind Randomized Control Trial. *Sci. Rep.* **6**, 36592; doi: 10.1038/srep36592 (2016).

**Publisher's note:** Springer Nature remains neutral with regard to jurisdictional claims in published maps and institutional affiliations.

## Figures and Tables

**Table 1:** Baseline characteristics of study participants.

Variables	Standard n (%)	Treatment n (%)	P value*
**Gender**			
Male	21 (47.7)	24 (54.5)	0.59
Female	23 (52.3)	20 (45.5)	
**Age (±SD)**	54.0 (±10.2)	53.7 (±10.8)	
<30			
31–40	4 (9.1)	7 (15.9)	0.41
41–50	11 (25)	7 (15.9)	
>50	29 (65.9)	30 (68.2)	
**Co-Morbid**			
Diabetes	31 (70.5)	28 (63.6)	0.54
**Race**			
Malay	43 (97)	43 (97)	—
**Marital status**			
Single	1 (3)	1 (3)	—
Married	43 (97)	43 (97)	
**Dialysis years**			
1	11 (25.0)	10 (22.7)	0.84
2–3	19 (43.2)	17 (38.6)	
3–4	8 (18.2)	8 (18.2)	
4–5	2 (4.5)	5 (11.4)	
>5	4 (9.1)	4 (9.1)	
**Smoking**			
Current smoker	11 (25)	9 (20.5)	0.05
Ex-smoker	2 (4.5)	10 (22.7)	
Never	31 (70.5)	25 (56.8)	
**Education status**			
No formal education	3 (6.8)	11 (25)	0.05
Primary	31 (70.5)	22 (50)	
Secondary	8 (18.2)	10 (22.7)	
Territory	2 (4.5)	1 (2.3)	
**Socio-economic condition**			
Low	5 (6.8)	15 (34.1)	0.01
Middle	31 (70.5)	23 (59.1)	
High	8 (18.2)	3 (6.8)	
**Employment Status**			
Employed	7 (15.9)	9 (20.5)	0.61
Unemployed	6 (13.6)	10 (22.7)	
Retried	12 (27.3)	11 (25.0)	
House wife	19 (43.2)	14 (31.8)	
**Medication**			
Alpha Antagonist	12 (27.2)	7 (15.9)	0.28
Beta Antagosnist	8 (18.1)	12 (27.2)	0.82
ARB	0	45 (100)	—
Ace Inhibitor	0	0	—
Calcium channel blocker	37 (84.1)	32 (72.7)	0.21
Diuretic	16 (36.3)	13 (29.5)	0.01
Statin	33 (75)	32 (71.7)	0.85
**Number of medications**			
1	9 (20.4)	1 (2.3)	0.07
2	14 (31.8)	11 (25)	
3	16 (36.3)	22 (50)	
>3	5 (11.3)	10 (22.7)	

*Chi square, Angiotensin receptor blocker (ARB), angiotensin converting enzyme inhibitor (ACE Inhibitor).

**Table 2:** Base line values for both arms (n = 44 each arm) and after 8 weeks of follow up.

Variables	Baseline		Standard	Treatment	Standard	Treatment
	Standard (±SD)	Treatment (±SD)	8 week (±SD)	8 weeks (±SD)	Standardized Mean Difference (CI 95%)	Standardized Mean Difference (CI 95%)
Interdialytic weight gain	1.6 (±0.6)	1.9 (±1.1)	1.4 (±0.5)	1.5 (±0.5)	0.7 (0.09, 0.26)	0.14 (−0.12, 0.34)
Pre-dialysis systolic	167.5 (±18.2)	168.6 (±16.3)	164.3 (±13.5)	159.6 (±12.1)	0.35 (0.46, 6.10)	0.33 (0.82, 15.1)
Pre-dialysis diastolic	80.1 (±12.7)	81.9 (±12.5)	79.1 (±8.3)	79.4 (±9.8)	0.12 (−1.47, 3.45)	0.03 (−4.74, 6.02)
Intradialytic systolic	153.6 (±20.6)	148.9 (±29.3)	153.7 (±16.9)	151.9 (±17.4)	0.01 (−2.27, 2.07)	0.06 (−6.69, 10.1)
Intradialytic diastolic	78.1 (±11)	81.2 (±19.7)	76.7 (9.2)	76.4 (8.7)	0.3 (−0.01, 2.8)	0.11 (−2.73, 6.19)
Post dialysis systolic	157.5 (±14.3)	156.3 (±13.4)	157.2 (±13.9)	153.9 (±11.7)	0.61 (−0.63, 1.17)	0.17 (−2.81, 9.89)
Post dialysis diastolic	80.6 (±12.6)	80.7 (±9.7)	78.6 (±9.6)	78.1 (±8.2)	0.25 (−0.41, 4.46)	0.16 (−2.24, 7.56)

*Standard deviation (SD), confidence interval (CI).

**Table 3:** Simple linear regressions for patient factors related to drop in blood pressure at the end of 2 month follow up.

Variables	Treatment			Standard		
	B coefficient	95% CI	p value	B coefficient	95%CI	p value
**Gender**						
Male	0.37	130.9, 151.6	**<0.01**	0.07	−6.5, 10.6	0.63
Female						
**Age group**						
<30		—		—		
31–40	−0.18	−15.6, 3.8	0.22	−0.6	−19.6, 10.1	0.52
41–50	0.09	−7.0, 12.6	0.56	0.2	−2.9, 16.4	0.16
>50	0.07	−5.8, 9.6	0.62	−0.13	−12.8, 5.1	0.38
**Co-Morbid**						
Diabetes	−0.35	−15.6, −1.5	**0.01**	−0.3	−20.0, −2.6	**0.01**
**Marital status**						
Married	−0.19	−14.8, 33.3	0.44	0.1	−16.1, 40.9	0.38
Single						
**Dialysis years**						
1	−0.35	−18.17, −1.2	0.02	−0.1	−13.2, 6.4	0.49
2–3	0.23	−2.1, 13.3	0.15	−0.1	−11.4, 5.8	0.51
3–4	0.13	−5.6, 13.8	0.39	−0.1	−19.1, –8.5	0.4
4–5	0.05	−13.6, 9.94	0.74	−0.03	−12.2, 10.1	0.84
>5	−0.10	−16.7, 8.3	0.50	0.3	3.4,−31.3	**0.01**
**Smoking**						
Current smoker	−0.12	−12.3, 5.4	0.43	0.04	−11.3, 8.4	0.76
Ex-smoker	−0.22	−14.6, 2.1	0.14	0.3	0.6, 39.8	**0.04**
Never	0.28	−0.2, 13.7	0.06	−0.09	−12.2, 6.4	0.53
**Education status**						
No formal education	0.20	−2.6, 13.6	0.18	0.05	−19.0, –23.1	0.8
Primary	−0.08	−7.4, 7.1	0.96	−0.1	−14.0, 20.0	0.72
Secondary	−0.29	−16.3, 0.2	0.06	0.1	−14.4, 4.1	0.26
Territory	0.24	−4.3, 42.7	0.10	0.2	−0.8, 38.6	0.06
**Socio-economic condition**						
Low	0.13	−4.3, 10.7	0.39	−0.1	−19.5, 7.2	0.3
Middle	−0.01	−7.6, 7.1	0.7	0.07	−7.2, 11.5	0.07
High	−0.21	−24.1, 3.8	0.15	−0.03	−9.9, 12.2	0.9
**Medication**						
Alpha Blocker	0.009	−9.6, 10.7	0.95	−0.09	−12.5, 6.6	0.54
Beta Blocker	0.17	−3.3, 12.6	0.24	0.09	−6.3, 11.9	0.54
Calcium channel blocker	−0.13	−11.6, 4.4	0.37	0.02	−10.8, 12.6	0.87
Diuretic	0.24	−1.6, 13.8	0.11	0.03	−7.5, 9.7	0.87

*Confidence interval (CI).

**Table 4:** Post dialysis systolic blood pressure difference from baseline to study duration among both the groups.

Groups	Variable	Mean (standard error)	Ranks N		Cohen’s d	p value
			Negative	Positive		
Standard group	Baseline systolic	157.5 (2.1)				
	8 week systolic	157.2 (2.1)	19	24	0.09	0.75
Treatment group	Base line systolic	156.3 (2.0)				
	8 week systolic	153.9 (1.7)	38	6	0.43	<0.001

*Wilcoxon test.

**Table 5:** Adverse events during patient follow-up.

Variables	None	Standard		>5	Treatment		3–5	>5	P value
		≤2	3–5		None	≤2			
**Inter-dialysis**									
Chills		1				1			—
hypotension					—				
Headache		12				10	2		0.34
**Post-dialysis**									
Chills		3			—				—
hypotension					—				—
Headache									—
Shortness of Breath		2				5			0.16
Dizziness					—				—
Leg cramps		11				13		1	0.39
Hyperkalemia					—				

*Chi-square.
